# Depth-Resolved OCT of Root Canal Walls After Diode-Laser Irradiation: A Descriptive Ex Vivo Study Following a Stereomicroscopy Report

**DOI:** 10.3390/diagnostics15233083

**Published:** 2025-12-04

**Authors:** Adrian Ştefan Stănuşi, Oana Andreea Diaconu, Andreea Stănuşi, Eugen Osiac, Mihaela Roxana Brătoiu, Gabriel-Sebastian Petrescu, Adelina Smaranda Bugălă, Bogdan Dimitriu, Mihaela Jana Ţuculină

**Affiliations:** 1 Department of Endodontics, Faculty of Dental Medicine, University of Medicine and Pharmacy of Craiova, 200349 Craiova, Romania; adrian.stefan.stanusi@gmail.com (A.Ş.S.); oana.diaconu@umfcv.ro (O.A.D.); preda.smaranda@yahoo.com (A.S.B.); mtuculina@yahoo.com (M.J.Ţ.); 2 Department of Prosthodontics, Faculty of Dental Medicine, University of Medicine and Pharmacy of Craiova, 200349 Craiova, Romania; 3 Department of Biophysics, University of Medicine and Pharmacy of Craiova, 200349 Craiova, Romania; eugen.osiac@umfcv.ro; 4 Department of Oral and Maxillofacial Surgery, Faculty of Dental Medicine, University of Medicine and Pharmacy of Craiova, 200349 Craiova, Romania; sebigabriel_petrescu@yahoo.com; 5 Department of Endodontics, Faculty of Dentistry, University of Medicine and Pharmacy Carol Davila Bucharest, 050474 Bucharest, Romania; bogdan.dimitriu@umcfcd.ro

**Keywords:** diode laser, endodontics, optical coherence tomography, root dentin, thermal injury, diagnostic imaging

## Abstract

**Background/Objectives**: Diode lasers are used as adjuncts for endodontic disinfection, but their depth-resolved effects on root dentin are insufficiently described. This ex vivo study used optical coherence tomography (OCT) to qualitatively document laser-related morphological signatures on canal walls. **Methods**: Palatal roots from extracted maxillary first molars were standardized and hemisectioned to create specimens allocated to a conventional diode-laser protocol, a higher-power protocol, or control. A 940-nm diode laser with endodontic tips was applied per group. Swept-source OCT acquired serial B-scans along the root length. Two endodontists reviewed images for thermally induced morphological alterations (TIMAs). Reporting is descriptive. **Results**: OCT revealed laser-related hyper-reflective linear/radial signatures extending from the canal lumen toward the external root surface in laser-treated specimens. Qualitatively, signatures appeared more conspicuous and extended deeper with the higher-power protocol than with the conventional protocol. Findings were most evident in the coronal/middle thirds. Control specimens served to contextualize background appearances from preparation and sectioning. Representative B-scans illustrate typical patterns. The novelty of the present study results from the identification of areas of morphological alteration through the OCT examination of the walls of the root canals. **Conclusions**: Depth-resolved OCT can visualize dentinal alterations associated with diode-laser irradiation in an ex vivo model. These observations support careful parameter selection and motivate in situ studies with concurrent temperature monitoring and histologic correlation.

## 1. Introduction

Humanity’s fascination with the properties of light and its use in medicine dates back to ancient times. Developments in physics in the early 20th century laid the foundations for the laser theory developed by Albert Einstein [1], culminating in the invention of this special form of light in 1960 [2]. Shortly thereafter, researchers began to explore the possible application of laser technology in medicine and dental treatments [2,3].

The rapid development of laser technology together with the increasing understanding of laser-tissue interaction has allowed the expansion of the spectrum of possible uses of lasers in endodontics. After the completion of research in the field and the clinical use of these devices, the indications for lasers in endodontics have focused on laser irradiation of the endodontic system for its decontamination [4].

Laser irradiation has been accepted as an adjunct to conventional endodontic therapy due to its ability to decontaminate [5,6]. The diffusion of laser light into dentin makes it possible to eliminate microorganisms from portions of the root canals that are not accessible to conventional irrigating solutions [7]. The diode laser has a photothermal antibacterial effect on accessible bacteria, but also on inaccessible bacteria, whose cell death does not occur immediately. The photodestructive effect causes a significant bacterial alteration, with inhibition of cell growth, damage to the integrity of the cell wall, and accumulation of proteins [8].

The attention of researchers has focused both on the beneficial effects of laser irradiation of the endodontic system, as well as on the limitations of this technology and possible side effects, especially the increase in local temperature. If the local increase in tissue temperature exceeds the accepted limit, undesirable side effects occur that influence the outcome of endodontic treatment. Researchers have evaluated the temperature developed in dental tissues during laser irradiation of root canals by several investigation methods, especially by applying thermocouples and finite element analysis (FEA) [9,10,11,12]. In addition to these studies, research has been carried out using medical imaging methods, especially scanning electron microscopy (SEM), to assess the effects of local temperature increase on dental tissues [13,14,15,16,17,18,19,20,21]. Fewer studies have used stereomicroscopes/stereoscopes and optical coherence tomography (OCT) for medical investigation.

OCT is a non-invasive and non-radioactive imaging method that generates high-resolution images of the examined tissues [22,23]. The OCT system is based on a Michelson interferometer and measures the intensity of light reflected by the examined tissue, as well as the time interval required for the reflection to return [24,25]. OCT has been used in endodontic studies to identify root fractures [26,27,28,29,30], the degree of root canal cleaning [30], and the quality of root fillings [23,31,32,33].

The present study is a continuation of a previously published study by the authors [34], in which stereomicroscopic examination of the internal root canal walls after chemomechanical instrumentation and laser irradiation was performed. The objective of this study was to evaluate the appearance of those root walls by OCT examination and to correlate the aspects identified by the two examination methods, depending on the laser irradiation protocol applied.

## 2. Materials and Methods

### 2.1. Study Design and Ethical Approval

This was a comparative ex vivo study on extracted human maxillary first molars, designed to evaluate morphological alterations of root canal walls after diode-laser irradiation using optical coherence tomography (OCT). The protocol was approved by the Ethics Committee of the University of Medicine and Pharmacy of Craiova, Romania (No. 28/24.02.2021). Written informed consent for tooth extraction, study participation, and data publication was obtained from all donors.

### 2.2. Teeth, Eligibility Criteria, and Sample Preparation

Thirty-five upper first molars with intact palatal roots were collected, yielding 35 palatal root samples. From these samples, seventy hemisectioned palatal-root specimens (two per tooth) were obtained as described below.

Inclusion criteria: indication for extraction due to non-restorable caries and/or periodontal destruction; no pulpal pathology; intact palatal roots after extraction; donor consent.

Exclusion criteria: previous root canal treatment; full-coverage/prosthetic restorations; root fracture or external resorption. Teeth were extracted atraumatically, disinfected in 10% hydrogen peroxide for 10 min, ultrasonically scaled, and brushed. All screening and preparation were performed by two dentists (coordinator and operator) under a dental operating microscope (Prima Mμ DNT, Labomed, Los Angeles, CA, USA).

### 2.3. Chemo-Mechanical Preparation

Root canals were prepared chemo-mechanically using an E-Connect S endomotor (Eightteeth, Changzhou, China) with the Reciproc Blue system (VDW GmbH, Munich, Germany). Irrigation employed 5.25% sodium hypochlorite, 0.9% sodium chloride, and 17% EDTA, activated ultrasonically with an Ultra-X Activator (Eightteeth, Changzhou, China).

### 2.4. Study Groups and Laser Irradiation Protocols

The 35 upper first molars were assigned to three groups according to the diode-laser protocol applied to the palatal root canal:

- **Group A (Conventional protocol)**: 15 teeth; diode-laser irradiation performed as per recommended clinical settings.
- **Group B (Higher-power protocol)**: 10 teeth; diode-laser irradiation performed at increased power and without pauses between cycles.
- **Group C (Control)**: 10 teeth; no diode-laser irradiation applied.

Laser procedures were delivered with a 940-nm diode laser (Biolase, Foothill Ranch, CA, USA) using endodontic tips with a diameter of 200 μm (E2-20 EZ Tip; E2-14 EZ Tip). The tips were introduced in the palatal root canals at 1 mm distance from the apex. For teeth in group A, the laser power was set for 1 W in continuous wave. The teeth were subjected to 3 irradiation cycles, separated by 10 s breaks. This laser irradiation protocol is known as “conventional laser endodontics” [4].

For teeth in group B, the laser power was set for 2 W in continuous wave. The teeth from this group were subjected to 3 irradiation cycles, without breaks. This high-power protocol was selected to emphasize the laser effects on dental tissues when the parameters exceed the ones recommended by specialists.

For each tooth, the laser was activated while moving the endodontic tips helically from apical to coronal, with a speed of 2 mm/s, to avoid stationary heating [4].

Teeth were assigned to the three groups (A, B, or C) consecutively as they were prepared, following a randomized convenience allocation method.

Since the total number of teeth included in this study could not be divided equally into 3 groups and the aim of this study was to examine the stereomicroscopic and OCT appearance of the internal root walls, it was decided to distribute a larger number of teeth in group A.

For standardization, palatal roots were sectioned to a length of 10 mm measured from the apex using diamond burs with air-turbine and water cooling. Each palatal root was then split longitudinally into two halves (per Akhtar’s protocol) [35] and fixed in high-consistency silicone (Zetaplus L Intro KIT, Zhermack, Badia Polesine, Italy), yielding 70 specimens.

### 2.5. OCT Imaging Acquisition

**Figure 1:**
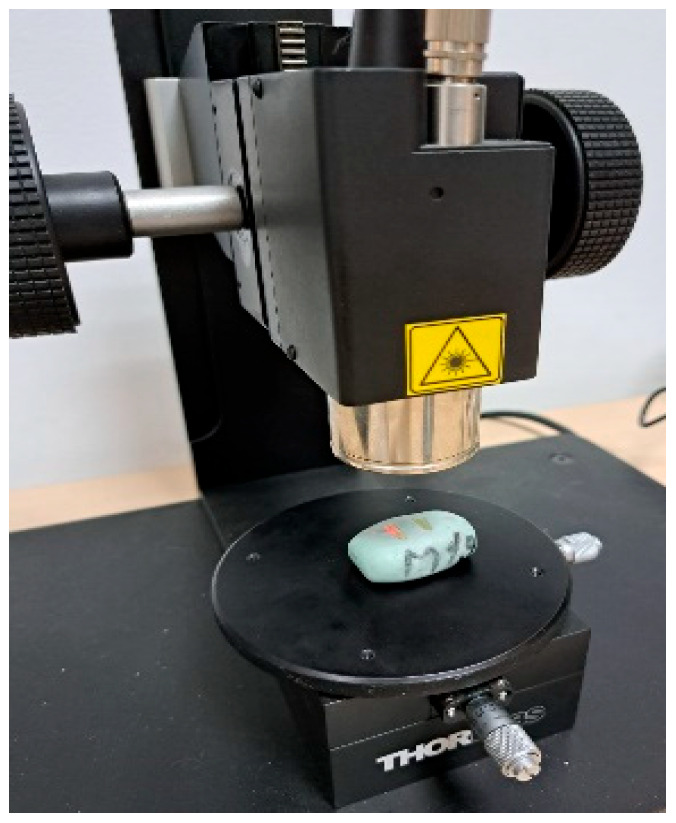
The OCT device was used to examine the samples.

**Figure 2:**
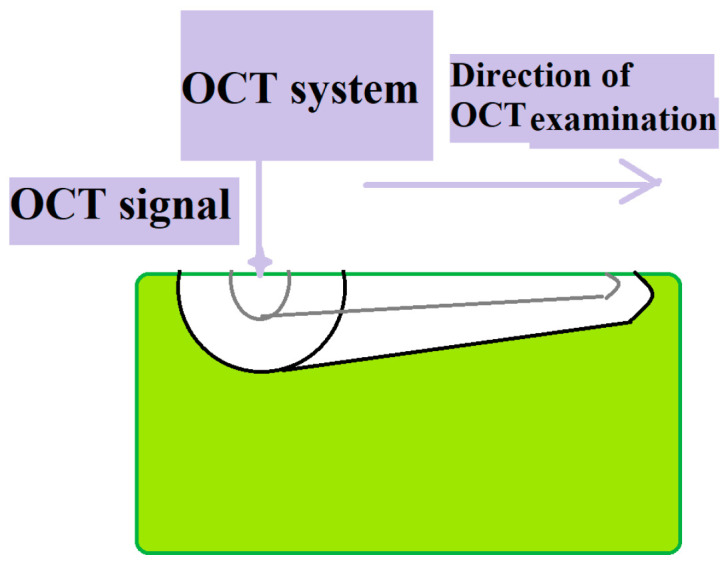
Schematic of OCT examination of samples.

Specimens were examined using a swept-source OCT system (OCS1300SS, Thorlabs, Craiova, Romania) with a center wavelength of 1310 nm, spectral bandwidth of 100 nm, and mean output of 12 Mw (Figure 1). OCT scans covered 10 mm along the root length with a tissue penetration depth of 2.5 mm (Figure 2). An index of refraction n = 1.56 (average of dentin 1.54 and cementum 1.58) was used for depth calibration. For each specimen, approximately 500 2D B-scans were acquired and stored as JPG files. Imaging was performed by a biophysics specialist. The OCT operator was blinded to the group allocation during image acquisition.

### 2.6. Image Interpretation

Two endodontists independently reviewed the OCT images to identify thermally induced morphological alterations (TIMAs) characterized by high-intensity signals with linear/radial disposition from the canal lumen toward the external root surface. Non-thermal artefacts from sectioning or forceps marks at the cut surface were excluded a priori based on location and morphology. Disagreements were resolved by consensus. No formal inter-rater statistics were computed.

### 2.7. Outcomes

- **Primary outcome:** Presence of thermally induced morphological alteration on OCT (binary, per specimen; per coronal/middle/apical third).
- **Secondary outcomes:** frequency and topographic distribution of alterations; qualitative pattern descriptors; agreement with stereomicroscopy findings from a prior study.

### 2.8. Relationship to Prior Publication and Data-Overlap Statement

A stereomicroscopic assessment of surface alterations on the same set of specimens was reported previously by the authors. The present work introduces a new imaging modality (OCT) and analyzes depth-resolved morphological changes not addressed in the prior report. No text, figures, or images are reproduced; all OCT data and analyses are original to this manuscript. The earlier article is cited wherever methods overlap, and the differences between protocols and outcomes are explicitly described [34].

### 2.9. Analysis Plan

Given the exploratory and descriptive nature of this study, a formal sample-size calculation was not performed; a convenience sample based on the availability of extracted teeth meeting eligibility criteria was used.

The analysis was qualitative and descriptive, focused on identifying and characterizing the morphological patterns of TIMAs. As the primary goal was to establish a verified final dataset, the image review process emphasized consensus.

Disagreements between the two independent reviewers (as described in Section 2.6) were resolved through joint discussion to reach a final consensus decision, rather than computing formal inter-rater reliability statistics. All findings are reported narratively, summarizing the presence, typical appearance, and topographic location (coronal/middle/apical third) of the observed alterations. Representative OCT B-scans are provided to illustrate these qualitative findings. No formal hypothesis testing was conducted.

## 3. Results

### 3.1. Specimen Flow and Allocation

In total, 35 palatal roots of maxillary first molars were prepared for OCT examination. For each palatal root sample, two specimens were obtained after separation, called “tooth code-a” and “tooth code-b”, respectively. Thus, 70 specimens were examined, initially with the NIKON SMZ 745T stereomicroscope (University of Craiova, Craiova, Romania) [34], then by the OCT method.

During the stereomicroscopic examination of the specimens, surface aspects of the root canal walls were assessed: presence/absence of pulp content, presence/absence of dentinal detritus, and presence/absence of areas of structural alteration [article reference adi stereo].

**Figure 3:**
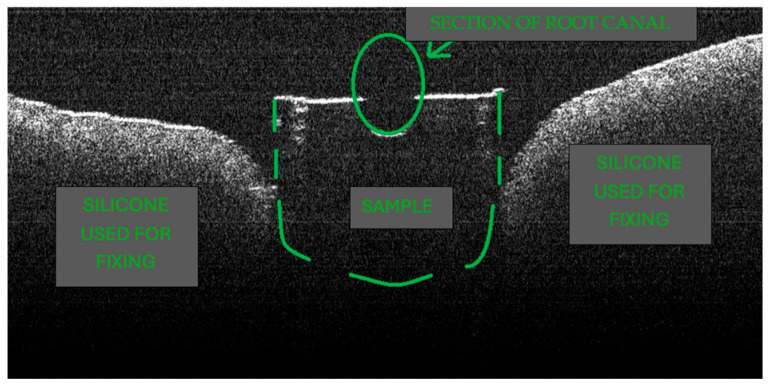
Schematic of the OCT image of an examined sample.

During the OCT examination, depth aspects of the root tissues were assessed. Figure 3 shows a schematic OCT image of an examined specimen.

Areas of structural alteration appear on OCT images as areas with high signal intensity, differentiated from the dentin mass and root cementum, and have different locations and appearances, depending on the determining factors.

### 3.2. Defining OCT Signatures: Alterations vs. Artefacts

**Figure 4:**
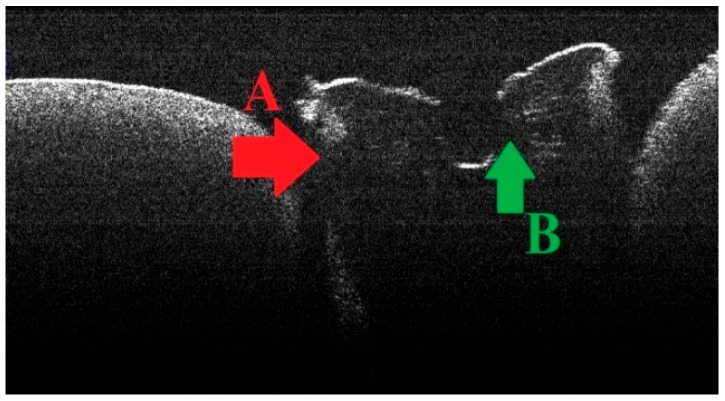
Schematic of the areas of morphological alteration: area A, indicated by the red arrow, marks the areas of alteration determined by root preparation; area B, indicated by the green arrow, marks the areas of alteration determined by laser irradiation.

For the areas of structural alteration at the root section surface and the area where the forceps jaws were applied, tissue damage occurred through stress accumulation and disruption of their continuity. Here, the areas of morphological alteration had an irregular shape and a higher intensity of the OCT signal on the OCT images (Figure 4—area indicated by the red arrow). The increase in signal intensity was the consequence of multiple reflections as a result of destructuring. These lesions were not located in the area of interest and were not taken into the study.

For the areas of structural alteration in the vicinity of the root canal, identified in some specimens belonging to teeth in groups A and B, tissue modification occurred through an increase in local temperature as a result of the laser irradiation technique. For these specimens, areas of irregular/radially arranged lines with increased OCT signal intensity were identified on the OCT images (Figure 4—area indicated by the green arrow). The increased signal intensity in these thermal lesions is a consequence of their increased refraction.

### 3.3. OCT Examination of Specimens from Group C

**Figure 5:**
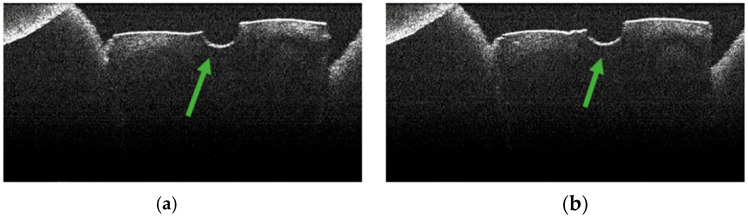
OCT images of specimen M28-a showing the empty root canal, indicated by the green arrow: (**a**) OCT image from the middle third; (**b**) OCT image from the apical third.

**Figure 6:**
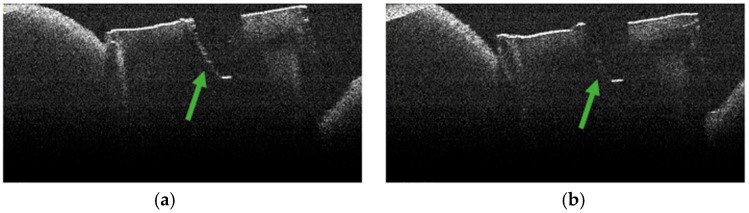
OCT images of specimen M33-b showing the empty root canal, indicated by the green arrow: (**a**) OCT image from the middle third; (**b**) OCT image from the apical third.

**Figure 7:**
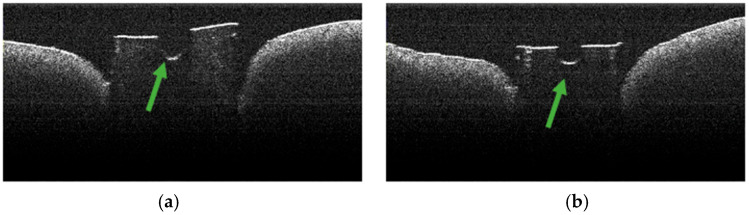
OCT images of specimen M35-a showing the empty root canal, indicated by the green arrow: (**a**) OCT image from the middle third; (**b**) OCT image from the apical third.

Control specimens exhibited background appearances related to preparation/sectioning without the characteristic laser-related signature pattern (Figure 5, Figure 6 and Figure 7).

OCT examination of these samples revealed the absence of pulpal content of the root canal, dentinal detritus, and areas of structural alteration of thermal origin. Only areas of structural alteration characteristic of the sample preparation method were identified on the OCT images obtained (Figure 5, Figure 6 and Figure 7).

Similarly, stereomicroscopic examination of these specimens did not reveal the presence of areas of morphological alteration [34].

### 3.4. OCT Examination of Specimens from Group A

**Figure 8:**
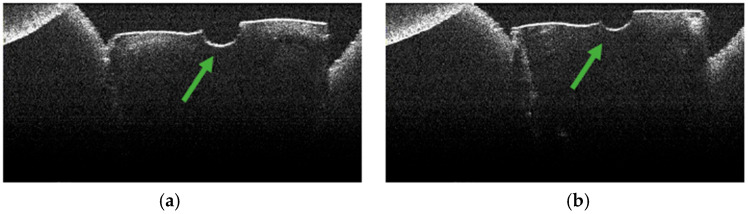
OCT images of specimen M5-a showing the empty root canal, indicated by the green arrow: (**a**) OCT image from the middle third; (**b**) OCT image from the apical third.

**Figure 9:**
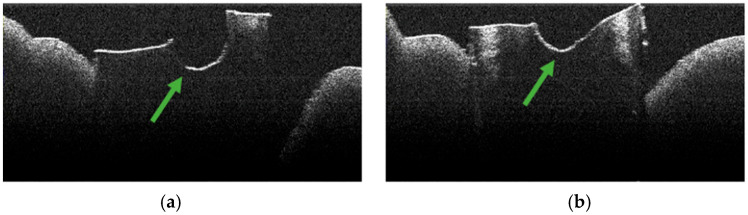
OCT images of specimen M3-a showing the empty root canal, indicated by the green arrow: (**a**) OCT image from the middle third; (**b**) OCT image from the apical third.

**Figure 10:**
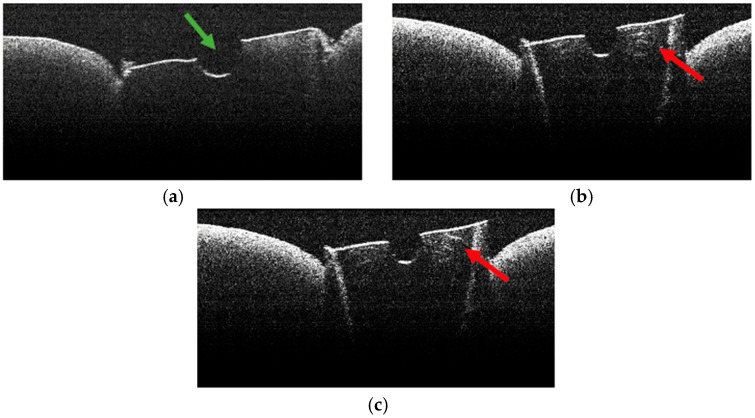
OCT images of specimen M11-a showing the empty root canal, indicated by the green arrow (**a**), and the lines of morphological alteration, indicated by the red arrows (**b**,**c**).

**Figure 11:**
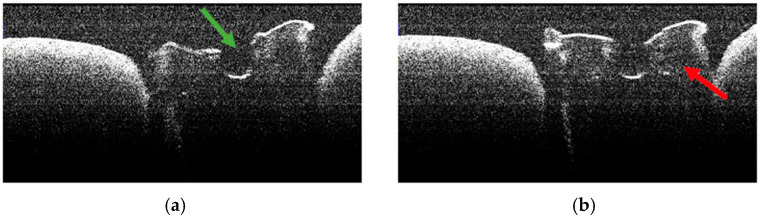
OCT images of specimen M9-b showing the empty root canal, indicated by the green arrow (**a**), and the lines of morphological alteration, indicated by the red arrows (**b**).

The absence of pulpal content and dentinal detritus was noted during the OCT examination of these specimens (Figure 8, Figure 9, Figure 10 and Figure 11). The same aspects were also noted during the stereomicroscopic examination of these specimens [34].

As the apical third was approached, in certain OCT images of several specimens, the existence of areas of morphological alteration was observed in the form of discrete light signals of greater intensity than the dentinal mass. These had the appearance of lines, with a radial arrangement, oriented from the root canal towards the external root surface (Figure 10b,c and Figure 11b).

The identified lines were interpreted as areas of morphological alteration of thermal origin. Their arrangement and location were noted, identical to those of the dentinal tubules. These lines of morphological alteration were not identified in the last portion of the apical third, corresponding to the area at which the endodontic laser tip was not activated.

Stereomicroscopic examination of these specimens, described in a previous publication [34], highlighted the existence of areas of morphological alteration, in the form of parallel white lines, interrupted by areas of normal appearance.

### 3.5. OCT Examination of Specimens from Group B

**Figure 12:**
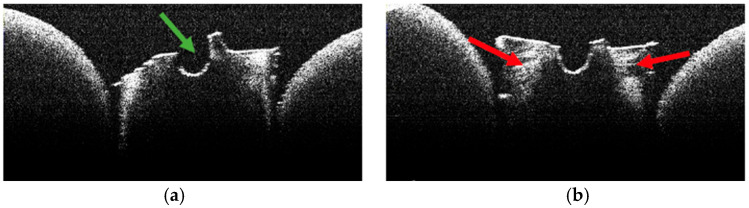
OCT images of specimen M16-a showing the empty root canal, indicated by the green arrow (**a**), and the areas of structural alteration of thermal origin, indicated by the red arrows (**b**).

**Figure 13:**
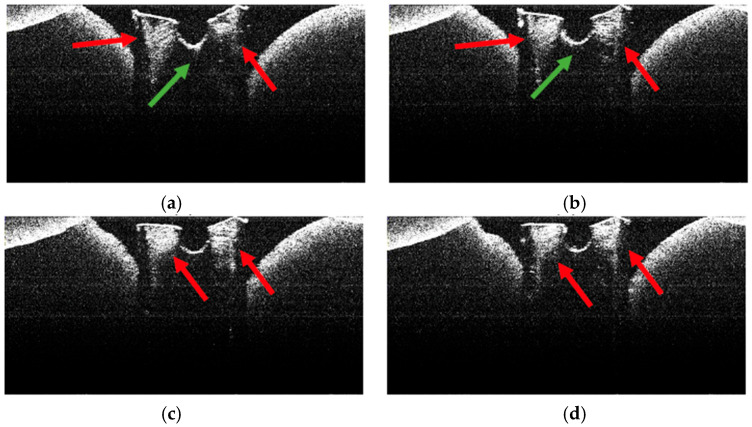
OCT images of specimen M17-a showing the empty root canal, indicated by the green arrow (**a**,**b**), and the areas of structural alteration of thermal origin, indicated by the red arrows (**a**–**d**).

**Figure 14:**
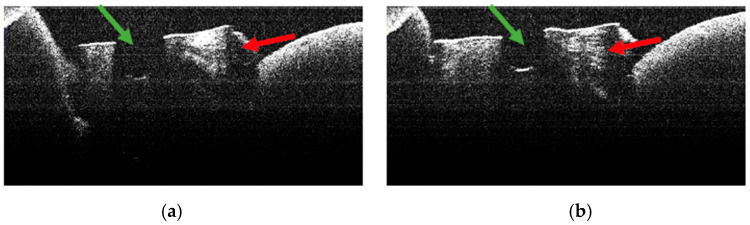
OCT images of specimen M20-b showing the empty root canal, indicated by the green arrow (**a**), and the areas of structural alteration of thermal origin, indicated by the red arrows (**b**).

**Figure 15:**
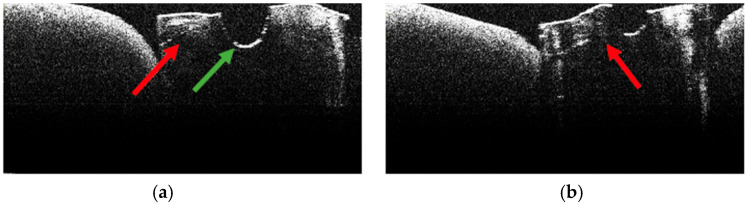
OCT images of specimen M25-b showing the empty root canal, indicated by the green arrow (**a**), and the areas of structural alteration of thermal origin, indicated by the red arrows (**a**,**b**).

The absence of pulpal content and dentinal detritus was also observed on OCT examination of these specimens (Figure 12, Figure 13, Figure 14 and Figure 15).

In most of the OCT images obtained, the existence of areas of structural alteration was noted, especially in the form of high-intensity light signals, with a linear appearance and radial arrangement, from the root canal to the external root surface (Figure 12, Figure 13, Figure 14 and Figure 15). Compared to Group A, these signals were considerably more frequent and more evident. It was appreciated that these changes have a thermal origin and occurred inside the dentinal tubules.

Similarly, on stereomicroscopic examination of these specimens, areas of morphological alteration, with irregular shapes and colored in white/brown, were observed [34].

In most specimens, OCT revealed prominent hyper-reflective signatures with a linear/radial pattern extending from the canal wall toward the external surface. Compared with Group A, these signatures appeared more frequent and more conspicuous. Examples are shown in Figure 13, Figure 14 and Figure 15.

### 3.6. Topographic Distribution

**Figure 16:**
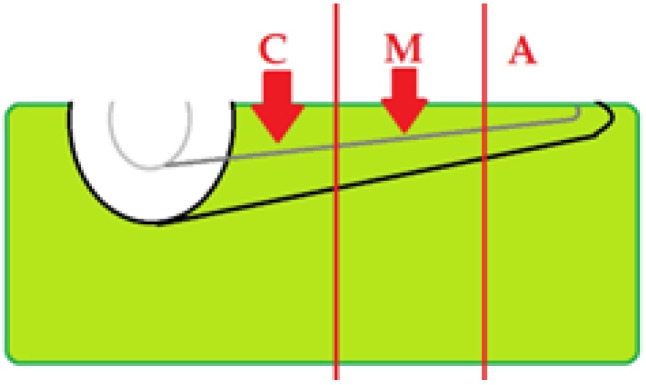
Schematic of OCT examination of specimens—Illustration of TIMAs localization at the level of thirds of the roots: C—coronal, M—middle, A—apical.

**Figure 17:**
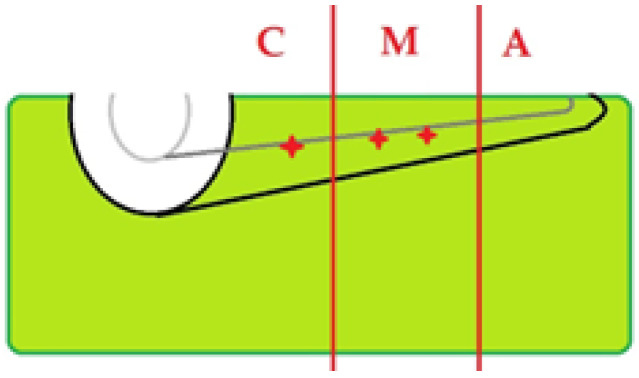
Schematic of OCT examination of specimens from group A. Illustration of TIMA localization.

**Figure 18:**
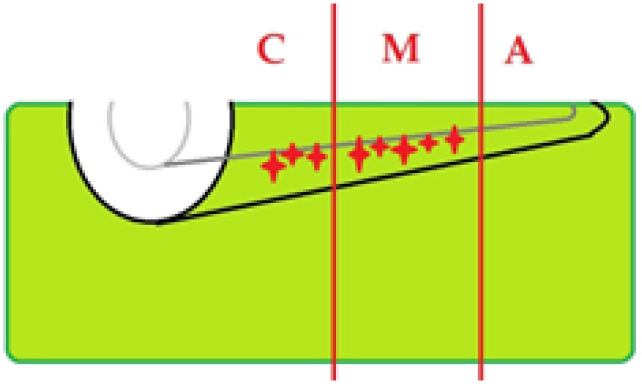
Schematic of OCT examination of specimens from group B. Illustration of TIMA localization.

Across laser-treated specimens, alterations were seen predominantly in the coronal and middle thirds, with occasional findings in the apical third (Figure 16). When present in Group A, signatures tended to be focal and limited (Figure 17), whereas Group B showed a broader distribution (Figure 18).

### 3.7. Frequency of TIMAs

For this study, the quantitative analysis was represented by the assessment of TIMAs per group of specimens.

For teeth in group A, TIMAs were identified in 4 specimens (out of 30), as shown in Table 1, representing 13.33% of all specimens from group A.

For teeth in group B, TIMAs were identified in 14 specimens (out of 20), as shown in Table 2, representing 70% of all specimens from group B.

## 4. Discussion

### 4.1. Interpretation in Context

OCT provides cross-sectional, high-resolution imaging of dentin and cementum and can visualize radial, hyper-reflective lines compatible with subsurface structural change. The present observations align with prior stereomicroscopy on the same specimen set while extending the assessment to the depth dimension not accessible to surface inspection. Where observed, focal apical-third signatures in Group A may reflect brief tip-wall contact or localized heat accumulation; the broader distribution in Group B is consistent with higher thermal load.

Laser irradiation of the endodontic system has been accepted as an adjunct to conventional endodontic therapy due to its decontamination ability [5,6]. The diffusion of laser light into dentin makes it possible to eliminate microorganisms from portions of the root canals that are not accessible to conventional irrigation solutions [7].

The user of the dental laser must take into account the thermal effects that develop in biological tissues when using a high-power device and be aware of the factors on which they depend. Probably the most important drawback of laser therapy is the thermal damage to the irradiated tissue as a result of the photothermal effect. The heat developed during laser-assisted endodontic techniques is dependent on the parameters of the laser device [33].

During dental laser irradiation of the root canal, a local increase in temperature occurs with the possibility of developing undesirable side effects related to the morphology of the root canal and the physical and chemical properties of the dental and periradicular tissues, but also to the settings of the laser device and the irradiation protocol used. In hard dental tissues, the increase in temperature can lead to the modification of the crystallographic characteristics of the mineral matrix, such as the crystalline skeleton, the size of hydroxyapatite crystals, and the formation of new compounds [33].

In a previous study, carried out using FEM, the temperature determined by conventional laser irradiation was measured, and it was assessed that it could not determine significant TIMA [10]. However, a similar study should be conducted using high-power protocol.

The heat developed in the root dentin during laser irradiation of the root canals determines the appearance of areas of morphological alteration. A process of fusion of the organic and inorganic dental structure occurs, followed by partial closure of the dentinal tubules [4,36]. Thus, the permeability of the root dentin decreases, and the adhesion of the sealers used for root obturation is compromised [37].

In the specialized literature, there are numerous studies that have evaluated the increase in temperature during the decontamination of root canals with diode lasers. The multitude of irradiation protocols applied and the variety of temperature measurement methods are noteworthy [9,12,38,39,40,41,42,43,44,45,46,47].

Significantly fewer studies have evaluated the morphological alterations that occur on the internal root wall, which can influence the quality of endodontic treatment, both in terms of root canal sterilization and the quality of root filling [34,36].

The present study is a continuation of a previously published study [34], which aimed to evaluate by stereomicroscopic examination the morphological changes that occur in the root dentin after irradiation with a diode laser with a wavelength of 940 nm. The objective of this study was to identify the morphological changes by OCT examination and to correlate the aspects identified by the two examination methods.

OCT provides images of sections of the examined tissue, obtained in a non-contact and non-invasive manner, in real time in situ, without the need for biopsy, histological procedures, or the use of X-rays [24,30].

As stated in the previous publication [34], for this research the palatal roots belonging to 35 upper first molars were used, due to the increased volume that facilitated obtaining the specimens. For the chemo-mechanical instrumentation of the palatal canals, a rotating file system was used, with a large taper, which facilitated the manipulation of the laser tip inside the root canals.

Examining the samples from group A by OCT, we noted the presence of areas of morphological alteration of thermal origin in several of them, being the same samples with alterations identified by stereomicroscopic examination [34]. Upon OCT examination, the alterations of thermal origin had the appearance of discrete lines with a radial disposition, arranged along the dentinal canals, with preferential localization in the apical third and a low frequency. We assessed that these developed in the dentinal canals as a result of the alteration of collagen and the fusion of organic and inorganic structures by increasing the local temperature, as described by specialists in the profile studies [4,36,37,47].

We consider that the morphological alterations identified in several samples from group A were determined by the direct contact of the endodontic tip with the walls of the root canals in their apical third. We consider that the small size of the alteration areas could not influence the seal of the future canal obturation.

Examining the OCT samples from group B, we noted the existence of morphological alterations of thermal origin for most of them. There were also the same samples from group B with alterations identified by stereomicroscopic examination [article stereo adi]. On the OCT images, the morphological alterations of thermal origin had, again, the appearance of lines with a radial arrangement along the dentinal tubules, but this time, compared to the OCT examination of the samples from group A, the increased signal intensity and the high frequency of these lesions were noted.

We considered that the morphological alterations identified in the samples belonging to group B were determined by the increase in local temperature above the limits accepted by the dental tissues. We considered that, due to their size, these areas of morphological alteration could influence the tightness of the future canal obturation.

OCT examination of samples from group C did not reveal areas of morphological alteration of thermal origin and served to compare and assess the aspects identified when examining samples from groups A and B.

Our study draws the attention of dental laser users to the importance of respecting the operative protocol for decontamination of root canals. Practitioners must be aware of the thermal effects that develop in dental and periodontal tissues during the use of the dental laser, as well as the long-term implications. The increase in tissue temperature causes changes in its structure, a fact also demonstrated by our study, which can influence the success of endodontic treatment.

The novelty of the present study results from the identification of areas of morphological alteration through the OCT examination of the walls of the root canals. To our knowledge, there are no studies in the specialized literature in the last 20 years that use the OCT method to examine the walls of the root canals after their conventional laser irradiation.

### 4.2. Strengths and Limitations

Strengths include standardized preparation, two independent readers with consensus, and depth-resolved imaging. Limitations include the ex vivo design, convenience sampling, lack of temperature monitoring and histologic correlation, and descriptive (non-inferential) reporting.

### 4.3. Practical Implications

The qualitative differences between protocols underscore the importance of parameter selection, tip motion (helical withdrawal), and respecting a non-activation apical buffer to mitigate heat concentration. OCT may serve as a complementary quality-assurance tool in preclinical protocol development.

## 5. Conclusions

OCT visualizes dentinal alterations associated with diode-laser irradiation in an ex vivo model. A higher-power protocol yields more frequent and conspicuous signatures than a conventional protocol, whereas controls lack these features. These qualitative observations support careful parameter selection and justify further in situ validation with thermal monitoring and histologic correlation.

## Figures and Tables

**Table 1:** Specimens from group A, with TIMA.

No.	Specimens with TIMA
1	M3-b
2	M9-b
3	M11-a
4	M15-b

**Table 2:** Specimens from group B, with TIMA.

No.	Specimens with TIMA
1	M16-a
2	M16-b
3	M17-a
4	M18-a
5	M19-a
6	M19-b
7	M20-a
8	M20-b
9	M22-b
10	M23-a
11	M23-b
12	M24-b
13	M25-a
14	M25-b

## Data Availability

The raw data supporting the conclusions of this article will be made available by the authors on request.

## Abbreviations

The following abbreviations are used in this manuscript:

OCT	Optical coherence tomography
TIMAs	Thermally induced morphological alteration
SEM	Scanning electron microscope
FEA	Finite element analysis

## References

[1] Einstein A. (1917). Zur Quantentheorie der Strahlung. Physiol. Z..

[2] Mady M., AlArabi A.A., Turkistani A.M., AlSani A.A., Murad G.S., AlYami A.S., Masmali A.M., AlGhamdi G.A., AlJohani E.H., AlKhuder M.S. (2022). The Role of Laser in Modern Dentistry: Literature Review. Ann. Dent. Spec..

[3] Convissar R.A. (2016). Principles and Practice of Laser Dentistry.

[4] Olivi G., De Moor R., Divito E. (2016). Lasers in Endodontics—Scientific Background and Clinical Applications.

[5] Anagnostaki E., Mylona V., Parker S., Lynch E., Grootveld M. (2020). Systematic Review on the Role of Lasers in Endodontic Therapy: Valuable Adjunct Treatment?. Dent. J..

[6] Kaplan T., Sezgin G.P., Sönmez Kaplan S. (2021). Effect of a 980-nm diode laser on post-operative pain after endodontic treatment in teeth with apical periodontitis: A randomized clinical trial. BMC Oral Health.

[7] Wenzler J.S., Falk W., Frankenberger R., Braun A. (2021). Impact of Adjunctive Laser Irradiation on the Bacterial Load of Dental Root Canals: A Randomized Controlled Clinical Trial. Antibiotics.

[8] Fahim S.Z., Ghali R.M., Hashem A.A., Farid M.M. (2024). The efficacy of 2780 nm Er,Cr;YSGG and 940 nm Diode Laser in root canal disinfection: A randomized clinical trial. Clin. Oral Investig..

[9] Haidary D., Franzen R., Gutknecht N. (2016). Root Surface Temperature Changes During Root Canal Laser Irradiation with Dual Wavelength Laser (940 and 2780 nm): A Preliminary Study. Photomed. Laser Surg..

[10] Stănuşi A.S., Popa D.L., Ionescu M., Cumpătă C.N., Petrescu G.S., Ţuculină M.J., Dăguci C., Diaconu O.A., Gheorghiţă L.M., Stănuşi A. (2023). Analysis of Temperatures Generated during Conventional Laser Irradiation of Root Canals—A Finite Element Study. Diagnostics.

[11] Al-Zand S.A., Al-Maliky M.A., Mahmood A.S., Al-Karadaghy T.S. (2018). Temperature elevation investigations on the external root surface during irradiation with 940 nm diode laser in root canal treatment. Saudi Endod. J..

[12] Mitic D., Cetenovic B., Jovanovic I., Gjorgievska E., Popovic B., Markovic D. (2017). Diode Laser Irradiation in Endodontic Therapy through Cycles—In vitro Study. Balk. J. Dent. Med..

[13] Suer K., Ozkan L., Guvenir M. (2020). Antimicrobial effects of sodium hypochlorite and Er,Cr:YSGG laser against Enterococcus faecalis biofilm. Niger. J. Clin. Pr..

[14] Abraham S., Vaswani S.D., Najan H.B., Mehta D.L., Kamble A.B., Chaudhari S.D. (2019). Scanning electron microscopic evaluation of smear layer removal at the apical third of root canals using diode laser, endo Activator, and ultrasonics with chitosan: An in vitro study. J. Conserv. Dent..

[15] Todea D.C.M., Luca R.E., Bălăbuc C.A., Miron M.I., Locovei C., Mocuţa D.E. (2018). Scanning electron microscopy evaluation of the root canal morphology after Er:YAG laser irradiation. Rom. J. Morphol. Embryol..

[16] Mohmmed S.A., Vianna M.E., Penny M.R., Hilton S.T., Mordan N., Knowles J.C. (2017). Confocal laser scanning, scanning electron, and transmission electron microscopy investigation of Enterococcus faecalis biofilm degradation using passive and active sodium hypochlorite irrigation within a simulated root canal model. MicrobiologyOpen.

[17] Cheng X., Xiang D., He W., Qiu J., Han B., Yu Q., Tian Y. (2017). Bactericidal Effect of Er:YAG Laser-Activated Sodium Hypochlorite Irrigation Against Biofilms of Enterococcus faecalis Isolate from Canal of Root-Filled Teeth with Periapical Lesions. Photomed. Laser Surg..

[18] Bao P., Shen Y., Lin J., Haapasalo M. (2017). In Vitro Efficacy of XP-endo Finisher with 2 Different Protocols on Biofilm Removal from Apical Root Canals. J. Endod..

[19] Cherian B., Gehlot P.M., Manjunath M.K. (2016). Comparison of the Antimicrobial Efficacy of Octenidine Dihydrochloride and Chlorhexidine with and Without Passive Ultrasonic Irrigation—An Invitro Study. J. Clin. Diagn. Res..

[20] Golob B.S., Olivi G., Vrabec M., El Feghali R., Parker S., Benedicenti S. (2017). Efficacy of Photon-induced Photoacoustic Streaming in the Reduction of Enterococcus faecalis within the Root Canal: Different Settings and Different Sodium Hypochlorite Concentrations. J. Endod..

[21] Latham J., Fong H., Jewett A., Johnson J.D., Paranjpe A. (2016). Disinfection Efficacy of Current Regenerative Endodontic Protocols in Simulated Necrotic Immature Permanent Teeth. J. Endod..

[22] Stănuşi A., Iacov-Crăițoiu M.M., Scrieciu M., Mitruț I., Firulescu B.C., Boțilă M.R., Vlăduțu D.E., Stănuşi A.Ş., Mercuț V., Osiac E. (2023). Morphological and Optical Coherence Tomography Aspects of Non-Carious Cervical Lesions. J. Pers. Med..

[23] Ţogoe M.M., Crăciunescu E.L., Topală F.I., Sinescu C., Nica L.M., Ioniţă C., Duma V.F., Romînu M., Podoleanu A.G., Negruţiu M.L. (2021). Endodontic fillings evaluated using en face OCT, microCT and SEM. Rom. J. Morphol. Embryol..

[24] Togoe M.M., Cojocariu A.C., Modiga C., Sinescu C., Duma V.F., Negruţiu M.L. (2019). Modern approaches of analysis and treatment of endodontic lesions using the endoscope and the optical coherence tomography. Rom. J. Oral Rehabil..

[25] de Oliveira B.P., Câmara A.C., Duarte D.A., Gomes A.S.L., Heck R.J., Antonino A.C.D., Aguiar C.M. (2017). Detection of Apical Root Cracks Using Spectral Domain and Swept-source Optical Coherence Tomography. J. Endod..

[26] Brady E., Mannocci F., Brown J., Wilson R., Patel S. (2014). A comparison of cone beam computed tomography and periapical radiography for the detection of vertical root fractures in nonendodontically treated teeth. Int. Endod. J..

[27] Chavda R., Mannocci F., Andiappan M., Patel S. (2014). Comparing the in vivo diagnostic accuracy of digital periapical radiography with cone-beam computed tomography for the detection of vertical root fracture. J. Endod..

[28] Yoshioka T., Sakaue H., Ishimura H., Ebihara A., Suda H., Sumi Y. (2013). Detection of root surface fractures with swept-source optical coherence tomography (SS-OCT). Photomed. Laser Surg..

[29] Shemesh H., van Soest G., Wu M.K., van der Sluis L.W., Wesselink P.R. (2007). The ability of optical coherence tomography to characterize the root canal walls. J. Endod..

[30] Negrutiu M.L., Sinescu C., Topala F.I., Nica L., Ionita C., Marcauteanu C., Goguta L., Bradu A., Dobre G., Rominu M. Root canal filling evaluation using optical coherence tomography. Proceedings of the Biophotonics: Photonic Solutions for Better Health Care.

[31] Todea C., Balabuc C., Sinescu C., Filip L., Kerezsi C., Calniceanu M., Negrutiu M., Bradu A., Hughes M., Podoleanu A.G. (2010). En face optical coherence tomography investigation of apical microleakage after laser-assisted endodontic treatment. Lasers Med. Sci..

[32] Negrutiu M.L., Sinescu C., Hughes M., Bradu A., Todea C., Balabuc C.I., Filip L.M., Podoleanu A.G. (2008). Root canal filling evaluation using optical coherence tomography. Biophotonics: Photonic Solutions for Better Health Care.

[33] Stănuşi A.Ş., Stănuşi A., Gîngu O., Diaconu O.A., Ţuculină J.M., Cumpătă N.C. (2024). Stereomicroscopic Aspects of Root Canal Walls after Conventional Laser Endodontics—A preliminary study. Rom. J. Oral Rehabil..

[34] Akhtar H., Naz F., Hasan A., Tanwir A., Shahnawaz D., Wahid U., Irfan F., Ahmed M.A., Almadi K.H., Alkahtany M.F. (2023). Exploring the Most Effective Apical Seal for Contemporary Bioceramic and Conventional Endodontic Sealers Using Three Obturation Techniques. Medicina.

[35] Gutiérrez-Corrales A., Rizcala-Orlando Y., Montero-Miralles P., Volland G., Gutiérrez-Pérez J.L., Torres-Lagares D., Serrera-Figallo M.A. (2020). Comparison of diode laser—Oral tissue interaction to different wavelengths. In vitro study of porcine periodontal pockets and oral mucosa. Med. Oral Patol. Oral Cir. Bucal.

[36] Bago I., Sandrić A., Beljic-Ivanovic K., Pažin B. (2022). Influence of irrigation and laser assisted root canal disinfection protocols on dislocation resistance of a bioceramic sealer. Photodiagnosis Photodyn. Ther..

[37] Beer F., Farmakis E.T., Kopic J., Kurzmann C., Moritz A. (2017). Temperature Development on the External Root Surface During Laser-Assisted Endodontic Treatment Applying a Microchopped Mode of a 980 nm Diode Laser. Photomed. Laser Surg..

[38] Hmud R., Kahler W.A., Walsh L.J. (2010). Temperature changes accompanying near infrared diode laser endodontic treatment of wet canals. J. Endod..

[39] Wang X., Sun Y., Kimura Y., Kinoshita J., Ishizaki N.T., Matsumoto K. (2005). Effects of diode laser irradiation on smear layer removal from root canal walls and apical leakage after obturation. Lasers Med. Sci..

[40] Higuchi N., Hayashi J.I., Fujita M., Iwamura Y., Sasaki Y., Goto R., Ohno T., Nishida E., Yamamoto G., Kikuchi T. (2021). Photodynamic Inactivation of an Endodontic Bacteria Using Diode Laser and Indocyanine Green-Loaded Nanosphere. Int. J. Mol. Sci..

[41] Walling J., Kirchhoff T., Berthold M., Wenzler J.S., Braun A. (2021). Impact of thermal photodynamic disinfection on root dentin temperature in vitro. Photodiagnosis Photodyn. Ther..

[42] Al-Karadaghi T.S., Gutknecht N., Jawad H.A., Vanweersch L., Franzen R. (2015). Evaluation of Temperature Elevation During Root Canal Treatment with DualWavelength Laser: 2780 nm Er,Cr:YSGG and 940 nm Diode. Photomed. Laser Surg..

[43] da Fonseca Alvarez A., Moura-Netto C., Daliberto Frugoli A., Fernando C., Aranha A.C., Davidowicz H. (2012). Temperature changes on the root surfaces of mandibular incisors after an 810-nm high-intensity intracanal diode laser irradiation. J. Biomed. Opt..

[44] Nagayoshi M., Nishihara T., Nakashima K., Iwaki S., Chen K.K., Terashita M., Kitamura C. (2011). Bactericidal Effects of Diode Laser Irradiation on Enterococcus faecalis Using Periapical Lesion Defect Model. ISRN Dent..

[45] Alfredo E.M.M., Sousa-Neto M.D., Brugnera-Junior A., Silva-Sousa Y.T.C. (2008). Temperature variation at the external root surface during 980-nm diode laser irradiation in the root canal. J. Dent..

[46] da Costa Ribeiro A., Nogueira G.E., Antoniazzi J.H., Moritz A., Zezell D.M. (2007). Effects of diode laser (810 nm) irradiation on root canal walls: Thermographic and morphological studies. J. Endod..

[47] Coluzzi D.J., Parke S.P.A. (2017). Lasers in Dentistry–Current Concepts.

